# Fractional SIR epidemiological models

**DOI:** 10.1038/s41598-020-77849-7

**Published:** 2020-11-30

**Authors:** Amirhossein Taghvaei, Tryphon T. Georgiou, Larry Norton, Allen Tannenbaum

**Affiliations:** 1 Mechanical and Aerospace Engineering, University of Calfornia, Irvine, CA 92697 USA; 2 Department of Medicine, Memorial Sloan Kettering Cancer Center, New York, NY 10021 USA; 3 Depts of Computer Science and Applied Mathematics and Statistics, Stony Brook University, Stony Brook, NY 11794 USA

**Keywords:** Epidemiology, Experimental models of disease

## Abstract

The purpose of this work is to make a case for epidemiological models with fractional exponent in the contribution of sub-populations to the incidence rate. More specifically, we question the standard assumption in the literature on epidemiological models, where the incidence rate dictating propagation of infections is taken to be proportional to the product between the infected and susceptible sub-populations; a model that relies on strong mixing between the two groups and widespread contact between members of the groups. We contend, that contact between infected and susceptible individuals, especially during the early phases of an epidemic, takes place over a (possibly diffused) boundary between the respective sub-populations. As a result, the rate of transmission depends on the product of fractional powers instead. The intuition relies on the fact that infection grows in geographically concentrated cells, in contrast to the standard product model that relies on complete mixing of the susceptible to infected sub-populations. We validate the hypothesis of fractional exponents (1) by numerical simulation for disease propagation in graphs imposing a local structure to allowed disease transmissions and (2) by fitting the model to the JHU CSSE COVID-19 Data for the period Jan-22-20 to April-30-20, for the countries of Italy, Germany, France, and Spain.

## Introduction

The classical SIR (Susceptible, Infectious, Recovered) model of infectious disease dynamics, and all subsequent multi-compartmental derivative models, are based on a model for the incidence rate that is taken almost universally in the form

1 $$\begin{aligned} r(t)=\beta I(t)S(t), \end{aligned}$$

where *I*(*t*), *S*(*t*) represent the size of infected and susceptible sub-populations. The proportionality factor $\beta$ is typically determined on a case-by-case basis. Thus, if *R*(*t*) represents the size of the recovered population, assuming that all individuals undergo full recovery and thereby the total population $S(t)+I(t)+R(t)$ remains constant, the most basic model for transmissions is in the form of the following system of equations, known as ***SIR model***,

$$\begin{aligned} \frac{d}{dt} S(t)&= - r(t) +\eta R(t), \\ \frac{d}{dt} I(t)&= r(t) -\alpha I(t), \\ \frac{d}{dt} R(t)&= \alpha I(t) -\eta R(t), \end{aligned}$$

where $\alpha$ is the recovery rate (with time constant of recovery $\tau :=1/\alpha$) and $\eta$ a parameter regulating the rate at which immunity is lost over time. See^1,18^ for all the details about the SIR model together with an extensive list of references. Multi-compartmental models that include infected but asymptomatic individuals, deceased, or exposed, have also been considered. However, throughout, the basic feedback that drives the infection, *r*(*t*), is invariably as in (1).

In departure from this well-studied SIR paradigm, we propose a *fractional SIR (****fSIR****) model* with rate

2 $$\begin{aligned} r(t)&=\beta I(t)^\gamma S(t)^\kappa , \end{aligned}$$

where one or, possibly, both sub-populations are scaled by exponents that are typically less than 1. The justification for such a model stems from the fact that, at least during the initial phase of an epidemic, infection propagates outwards from infected cells to the general population. In such a scenario, where for instance $S(t)\gg I(t)$ (much greater), the boundary of infected cells which would roughly account for most new infections, scales as a fractional power $\gamma <1$ of the area of the cells, hence $I(t)^\gamma$. In actuality, due to the diffusive nature of infection-propagation amongst the general population, the exponent is expected to be larger than 1/2, as it would be in the continuous limit when the boundary is a smooth curve. Moreover, at least in the early phases of an epidemic, the exponent of *S*(*t*), which is significantly larger than *I*(*t*) may turn out to be negligible.

The idea of using fractional exponents in growth models has been motivated from Norton–Simons–Massagué (NSM) model, a growth model of the form

3 $$\begin{aligned} \frac{dV(t)}{dt} =aV^\lambda (t)-b V(t), \end{aligned}$$

with origins in the 1950s^3^. This type of model was designed to describe the growth of biological organisms employing certain energy principles. In the model, the parameters *a* and *b* quantify anabolism (growth) and catabolism (death), respectively. Equation (3) may be interpreted as asserting that the net growth rate of an organism results from the balance of synthetic and degradative mechanisms. While the rate of the former process follows a law of allometry (i.e., the rate is proportional to the volume *V*(*t*) via a power function), the rate of the latter process scales linearly with *V*(*t*). It is important to note that the two special cases of (3), (1) power law $b=0$, and (2) second-type growth $\lambda =2/3$ have already been successfully applied to describe tumor growth^10,27^. The general case, $0<\lambda <1$, was introduced in^23^ to explain the self-seeding hypothesis. Moreover, an important geometrical interpretation was provided in^2,22^. In these works, the authors relate the exponent $\lambda =d/3$ to the fractional Hausdorff dimension of the proliferative tissue, where *d* denotes the fractal dimension of the tissue. Moreover, the model (3) has been derived mechanistically by linking tumor growth to metabolic rate and vascularization^12^.

Thus, in a similar spirit to the Norton–Simons–Massagué (NSM) model, herein, we recognize the geometric constraints imposed on disease propagation by the locality of transmissions around infectious cells. To this end, we seek to explain the origin of fractional power in (2) by (1) numerical experiments, and (2) fitting such models to data sets.

Specifically, with respect to (1), we postulate a discrete model where infection propagates over nodes of a network. The network, representing individuals, is not in general planar, yet it is immersed in ${\mathbb {R}}^2$. While (physically) neighboring nodes may be densely connected, precluding the graph from being planar, the likelihood of being connected depends on their physical distance. We observed that such models lead to fractional exponents in the incidence rate, in agreement with (2).

While the basic mechanism of propagation of pathogens, through contacts (edges) of a social network follows the extensive foundational work on epidemics over networks^5,13,19,^^20^, Chapter 16, our proposed network structure in which edges between nearby nodes are prevalent, renders a number of analytical techniques (such as the renormalization methods used in^19^) inapplicable—with graphs failing to display a tree-like structure as in random networks. Thus, the verification that the postulated contact structure leads to fractional exponents is at present empirical. It is anticipated that statistical analysis, e.g., along the lines of^14^, may be adapted to reflect the particular structure in the type of networks we consider and lead to more rigorous analytical results.

Regarding (2), we have numerically studied data that is available in^7^ on the recent COVID-19 epidemic. We focused on the dynamics of transmission in four countries (Italy, Germany, France, and Spain) during the early phases of the epidemic. For the specific datasets we considered, as we explained in the results, the exponent $\gamma$ in (2) ranges from about 0.6 to 0.8. Coincidentally, the values appear similar to empirically determined exponents of the NSM model.

we should note that after the initial submission of this work, it came to our attention that fractional powers in the incidence rate of SIR models have been considered in^17,24,29^ and going further back to^26^. The authors of^17^ postulated that a threshold level of viral concentration in the population may be needed before an epidemic takes off, and that such a mechanism could account for nonlinear incidence rates, especially with exponents $\gamma >1$ that lead to rich dynamical behavior studied in^17^. The authors in^29^ suggest an exponent slightly less than 1 (specifically 0.97) in their simulations, to model “the biases introduced by time discretization and the fact the force of infection is disproportionately small at higher densities”. In^24^, the authors consider heterogeneity in the transmission coefficient $\beta$ and, for a particular initial distribution of $\beta$, obtain an effective fractional model with exponents larger than 1. In contrast, in our work, the proposed mechanism suggests $\gamma <1$. Moreover, we have attempted to link the fractional exponents in the dynamics of the epidemic to the structure of the network and, specifically, to the interface between infected and susceptible subpopulations.

## Models of discrete transmissions

To provide insight and justification for our hypothesis on the validity of Eq. (2), we develop a discrete model for direct transmissions between individuals consisting of nodes (individuals) on a graph that captures contacts between them. In the present work, the graph is fixed, while in future work we plan to explore the possibility of time-varying links between nodes as well as modeling control actions, such as social-distancing, so as to study the effects of such mediation protocols.

### Model: probabilistic SIR on a graph

Consider a simple undirected graph of size *n* with adjacency matrix *A*, where $A_{ij} = 1$ if node *i* and *j* are connected, and $A_{ij}=0$ otherwise . The graph is used to model the spread of infection over a network of nodes representing individual people. Every node can be in one the three states $\{S,I,R\}$. We use $x_i(t) \in \{S,I,R\}$ to represent the state of node *i* at time $t\in \{0,1,2,3,\ldots \}$. We consider the unit of time to be one day. Here, $\{x_1(t),\ldots , x_n(t)\}$ evolves, as a Markov chain on $3^n$ states, according to the following transition probabilities dictating transition at the node level for $x_i(t)$ to $x_i(t+1)$,

4 $$\begin{aligned}&S \rightarrow I \quad \text {w.p.} \quad 1-(1-\beta )^{N^{(I)}_i(t)}\nonumber \\&I \rightarrow R \quad \text {w.p.} \quad \alpha \nonumber \\&R \rightarrow S \quad \text {w.p.} \quad \eta . \end{aligned}$$

Here, $\beta$ is the transmission rate, $\alpha$ is the recovery rate, and $\eta$ is the susceptibility rate (quantifying loss of immunity over time). The notation $N^{(I)}_i(t)$ stands for the number of the neighbors of node *i* that are infected at time *t*, i.e.

$$\begin{aligned} N_i^{(I)}(t) = \sum _{j=1}^n A_{ij} \mathbb {1}_{\{x_j(t)=I\}}, \end{aligned}$$

where $\mathbb {1}_{\{\cdot \}}=1$ when $\{\cdot \}$ holds and is 0 otherwise.

With regard to the structure of the graph, specifying contacts between individuals, we describe results considering the following options:

#### Two-dimensional grid-graph

We work out two rudimentary models where individuals (nodes) are placed on a 2-dimensional grid (vertex set)

$$\begin{aligned} {\mathcal {V}}:=\left\{ v_{ij}=(i,j)\mid i,j\in \{0,1,\ldots , N-1\}\right\} . \end{aligned}$$

**Figure 1:**
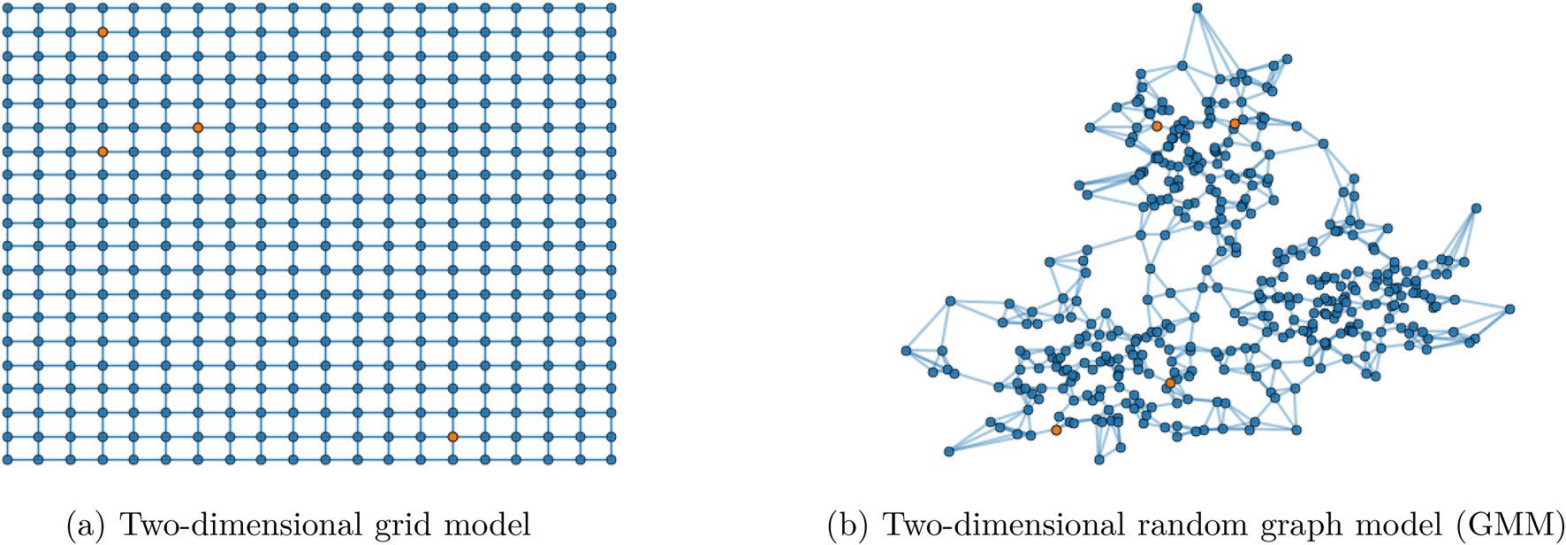
Structure of the two graph models considered in this paper. Nodes are connected to their 4 nearest neighbors. The initially infected people are marked in orange.

We carry out experiments for two cases, where the edge set is defined by

5 $$\begin{aligned} {\mathcal {E}}:=\left\{ (v_{ij},v_{\ell k}) \mid |i-\ell |+|j-k|\le d,\quad \forall i,j,l,k\in \{0,\ldots ,N-1\}\right\} , \end{aligned}$$

with $d\in \{1,2\}$. Thus, when $d=1$, each node is connected to $k=4$ nearest neighbors, while in the case where $d=2$ each node is connected to $k=8$ nearest neighbors (von Neumann neighborhood with Manhattan distances 1 and 2, respectively). The graph structure for the case $d=1$ is depicted in Fig. 1a. In either case, the infectious model is simulated and the results discussed in the section on experiments.

#### Two-dimensional random graph

We postulate a distribution of nodes on ${\mathbb {R}}^2$ according to a Gaussian mixture model (Fig. 1b). Each node is connected to its 4 nearest neighbor. Analogous conclusions are drawn and discussed in the section on experiments as well.

Table 1 includes summary of notation for modeling parameters.

We experiment with a randomized initialization, where a number of small initial cells of infected individuals are sprinkled randomly with probability $p_0$ inside the general population. A localized initialization, where a specified initial collection of neighboring nodes are infected and the contagion begins from these nodes, gives similar results.

We are interested in studying the dynamics of the subpopulations of susceptible, infected, and recovered individuals, denoted by *S*(*t*), *I*(*t*), and *R*(*t*),  respectively. These quantities are calculated from the state of the individuals $x_i(t)$:

$$\begin{aligned} \xi (t) = \sum _{i=1}^n\mathbb {1}_{\{x_i(t)=\xi \}}, \end{aligned}$$

for each $$\xi \in \{ S,I,R\}$$.

We model the change in the number of infected individuals $\Delta I(t) := I(t)-I(t-1)$ as a function of aggregate quantities *S*(*t*), *I*(*t*), and *R*(*t*). The change is mainly due to two factors, (1) an increase due to transmission of infection by contact between infected and susceptible individuals, denoted by $\Delta I_{\text {trans}}(t)$, and (2) a decrease due to recovery of individuals, denoted by $\Delta I_{\text {recovery}}(t)$. Thus,

6 $$\begin{aligned} \Delta I(t) = \Delta I_{\text {trans}}(t) - \Delta I_{\text {recovery}}(t). \end{aligned}$$

In terms of the state of the Markov chain, $\Delta I_{\text {trans}}(t)$ and $\Delta I_{\text {recovery}}$ are given by

$$\begin{aligned} \Delta I_{\text {trans}}(t)&:= \sum _{i=1}^n \mathbb {1}_{\{x_i(t)=I,x_i(t-1)=S\}},\\ \Delta I_{\text {recovery}} (t)&:= \sum _{i=1}^n \mathbb {1}_{\{x_i(t)=R,x_i(t-1)= I\}}, \end{aligned}$$

It is expected that $\Delta I_{\text {recovery}} (t) \approx \alpha I(t)$ on average. However, the model for $\Delta I_{\text {trans}}$ is not simple. We hypothesize the fractional model

$$\begin{aligned} \Delta I_{\text {trans}} \approx c I(t)^\gamma S(t)^\kappa . \end{aligned}$$

where *c* is a constant, and $\gamma$ and $\kappa$ are exponents. We test this hypothesis in the next section by carrying out Monte Carlo simulations on a network model and fitting the data to the proposed fractional model.

**Table 1:** Notation for modeling parameters.

Parameters	Definition
$\alpha$	Recovery rate
$\beta$	Transmission rate
$\eta$	Rate of losing immunity
$\kappa$	Fractional exponent of *S*
$\gamma$	Fractional exponent of *I*
*k*	Number of neighbours of each network node
*d*	Maximal Manhattan distance between neighbours
*m*	Number of additional random edges between randomly selected nodes

**Figure 2:**
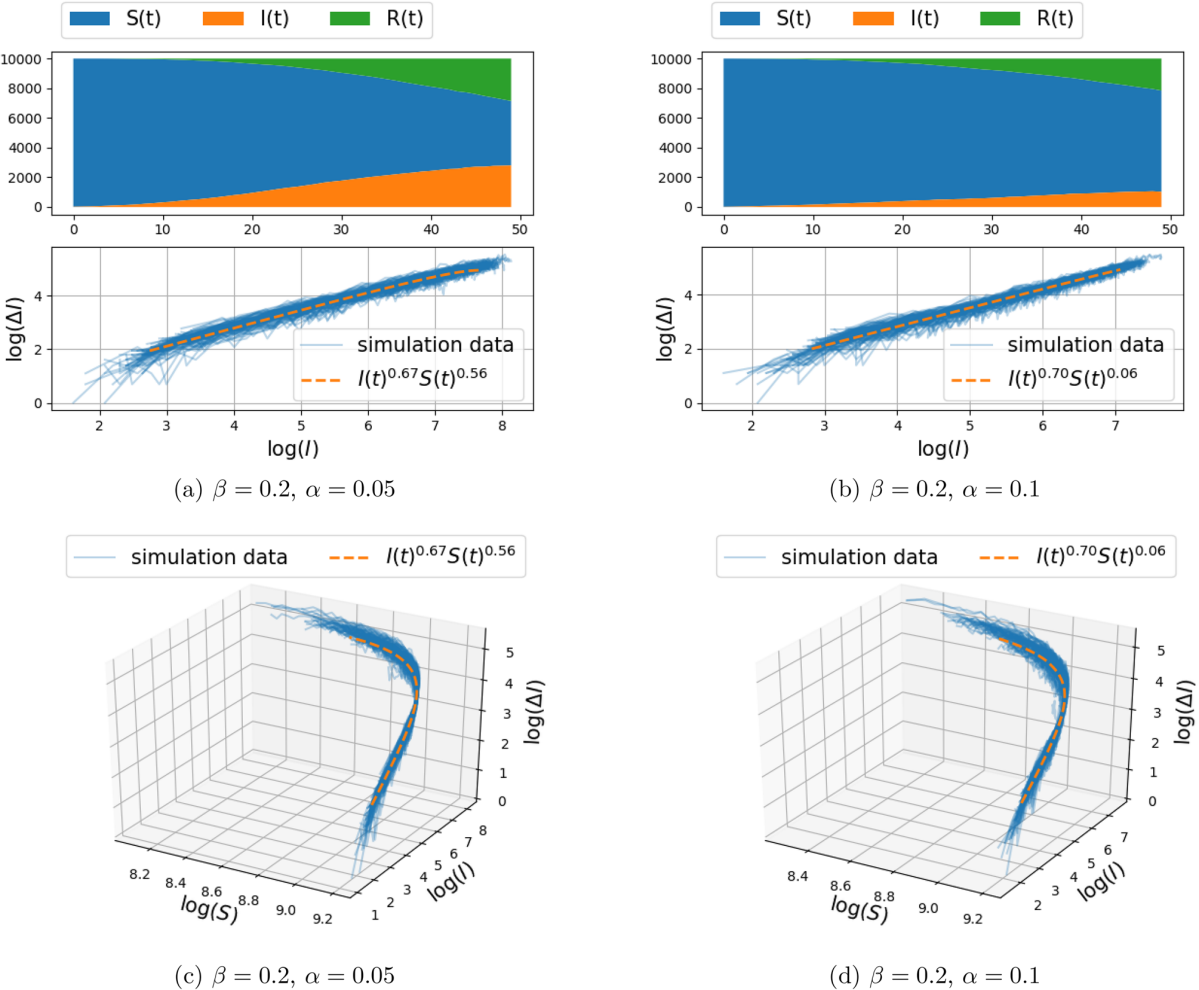
Spread of infection on a two-dimensional grid, with connection to $k=4$ nearest neighbors (i.e., $d=1$ in Eq. (5)) and transmission rate $\beta = 0.2$ (the recovery rate $\alpha$ is fixed to either 0.05 or 0.1). The top panels depict the number of susceptible, infected, and recovered individuals as a function of time over a range of 50 days, for a single realization of the stochastic model described in “Model: probabilistic SIR on a graph”. The center and bottom panel depict $(\Delta I_{\text {trans}}(t),I(t))$ and $(\Delta I_{\text {trans}}(t),I(t),S(t))$ respectively, for 100 Monte-Carlo simulations (with solid blue lines). The fractional model fit is illustrated with orange dashed curve. The fitting procedure maximizes the log-likelihood (9) over the free parameters. The value of the fitted exponents are shown in the legend.

**Figure 3:**
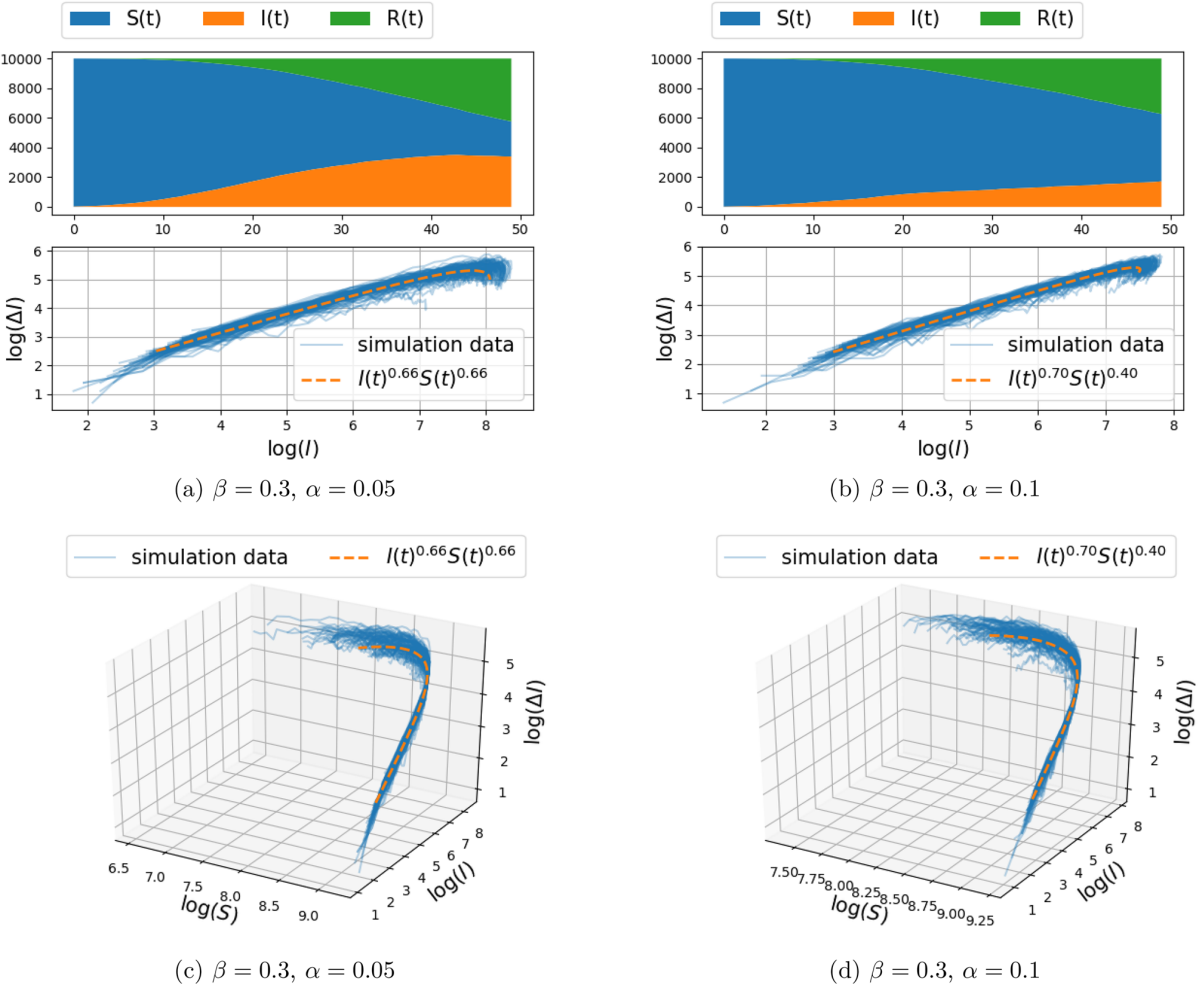
Spread of infection on a two-dimensional grid, with connection to $k=4$ nearest neighbors (i.e., $d=1$ in Eq. (5)) and transmission rate $\beta = 0.3$ (the recovery rate $\alpha$ is fixed to either 0.05 or 0.1). The top panels depict the number of susceptible, infected, and recovered individuals as a function of time over a range of 50 days, for a single realization of the stochastic model described in “Model: probabilistic SIR on a graph”. The center and bottom panel depict $(\Delta I_{\text {trans}}(t),I(t))$ and $(\Delta I_{\text {trans}}(t),I(t),S(t))$ respectively, for 100 Monte–Carlo simulations (with solid blue lines). The fractional model fit is illustrated with orange dashed curve. The fitting procedure maximizes the log-likelihood (9) over the free parameters. The value of the fitted exponents are shown in the legend.

## Experiments

### Two-dimensional grid-graph

**Figure 4:**
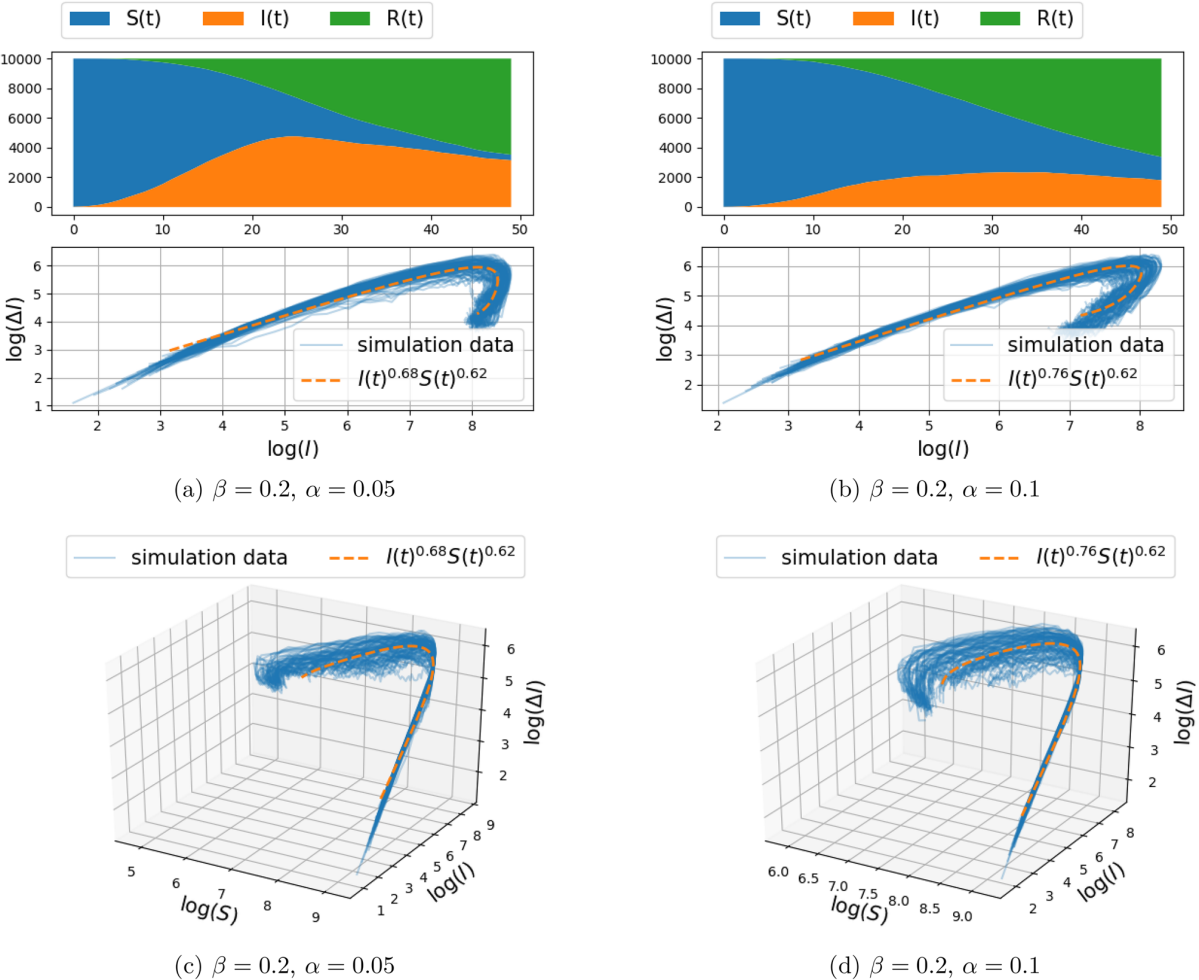
Spread of infection on a two-dimensional grid, with connection to $k=8$ nearest neighbors (i.e., $d=2$ in Eq. (5)) and transmission rate $\beta = 0.2$ (the recovery rate $\alpha$ is fixed to either 0.05 or 0.1). The top panels depict the number of susceptible, infected, and recovered individuals as a function of time over a range of 50 days, for a single realization of the stochastic model described in “Model: probabilistic SIR on a graph”. The center and bottom panel depict $(\Delta I_{\text {trans}}(t),I(t))$ and $(\Delta I_{\text {trans}}(t),I(t),S(t))$ respectively, for 100 Monte-Carlo simulations (with solid blue lines). The fractional model fit is illustrated with orange dashed curve. The fitting procedure maximizes the log-likelihood (9) over the free parameters. The value of the fitted exponents are shown in the legend.

**Figure 5:**
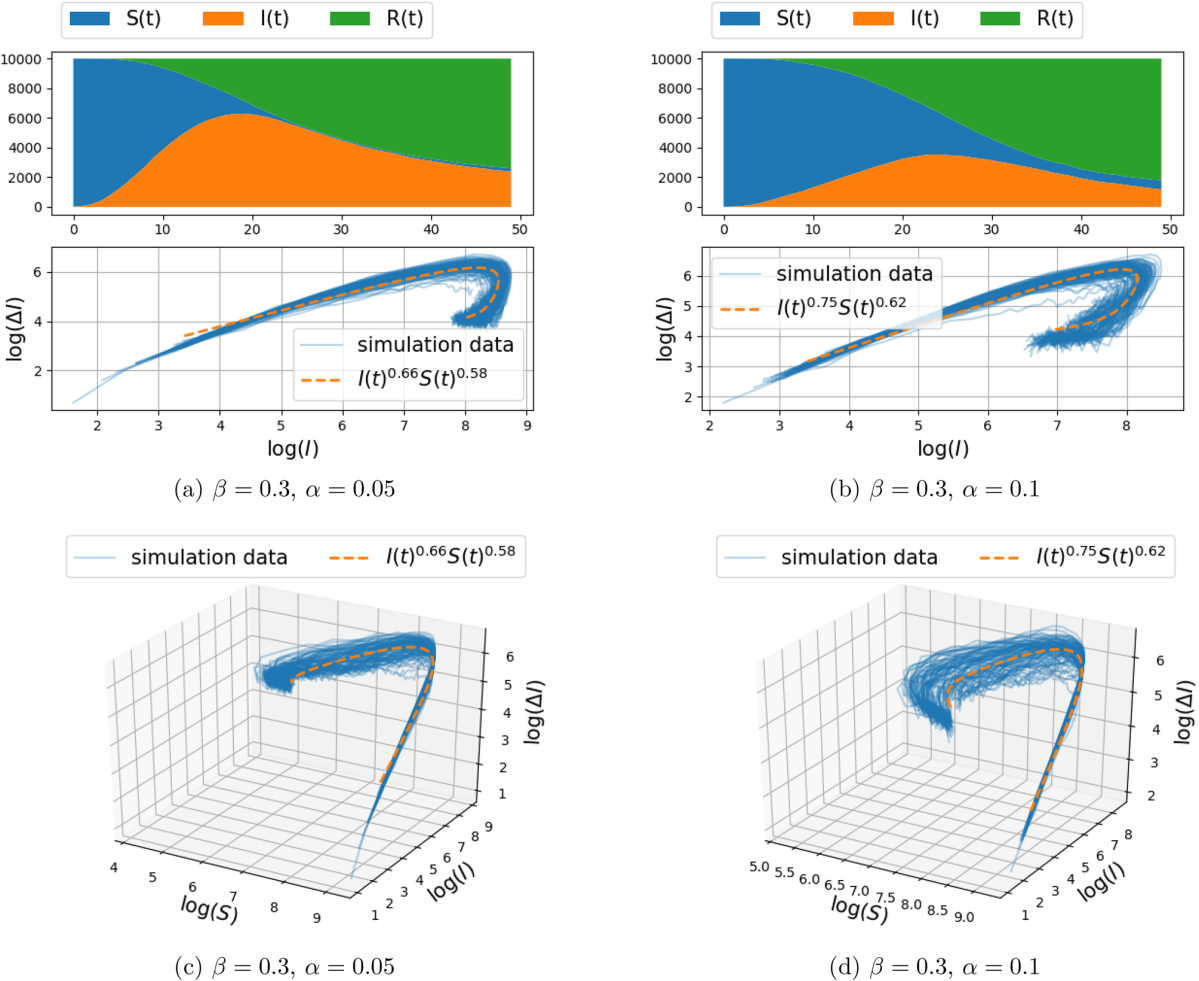
Spread of infection on a two-dimensional grid, with connection to $k=8$ nearest neighbors (i.e., $d=2$ in Eq. (5)) and transmission rate $\beta = 0.3$ (the recovery rate $\alpha$ is fixed to either 0.05 or 0.1). The top panels depict the number of susceptible, infected, and recovered individuals as a function of time over a range of 50 days, for a single realization of the stochastic model described in “Model: probabilistic SIR on a graph”. The center and bottom panel depict $(\Delta I_{\text {trans}}(t),I(t))$ and $(\Delta I_{\text {trans}}(t),I(t),S(t))$ respectively, for 100 Monte-Carlo simulations (with solid blue lines). The fractional model fit is illustrated with orange dashed curve. The fitting procedure maximizes the log-likelihood (9) over the free parameters. The value of the fitted exponents are shown in the legend.

Simulation results of infection spread on the two-dimensional grid-graph with size $100\times 100$, for $d\in \{1,2\}$ (and hence, each node connected to the $k\in \{4,8\}$ nearest nodes), were carried out for the model (4) and are presented in Figs. 2, 3 and Figs. 4, 5, respectively. The documented experiments were carried out for $\eta =0.01$, initial infection probability $p_0=10^{-3}$, combination of parameters for $\alpha =\{0.05,0.1\}$ and $\beta \in \{0.2,0.3\}$, and a time period of 50 days. In order to take into account the randomness of the stochastic model, 100 Monte Carlo simulations were carried out. Each simulation involves independent random initialization of the infected individuals, while the structure of the graph remains fixed.

The top panel in each figure depicts the number of susceptible, infected, and recovered individuals as a function of time, for a single realization of the stochastic model. The bottom panel (of the three panels) in each figure depicts in 3D the number of newly infected population due to transmission, $\Delta I_{\text {trans}}(t)$, vs. the number of infected people *I*(*t*), and the number of susceptible people *S*(*t*), with solid blue lines marking 100 independent simulations. The center panel represents the two-dimensional projection of the data points on the $(\log (\Delta I_{\text {trans}}(t)), \log (I))$ plane.

In order to capture the relationship between $\Delta I_{\text {trans}}(t)$ and *I*(*t*) and *S*(*t*), a parametric curve of the form $\Delta I_{\text {trans}}(t) = c I(t)^\gamma S(t)^\kappa$ is fitted to the data-points obtained from each simulation. Thus, the parameters *c*, $\gamma$, and $\kappa$ are obtained by a least-squares fit of the linear relation between respective logarithms (with zero entries for $\Delta I_{\text {trans}}(t),I(t),S(t)$ being ignored),

$$\begin{aligned} \log (\Delta I_{\text {trans}}(t))=\log (c)+\gamma \log (I(t))+\kappa \log (S(t)). \end{aligned}$$

The linear regression implicitly assumes an additive Gaussian perturbation, as in (8), on the log-transformed data. Such an additive perturbation of the logarithm is natural in population models as it ensures, here, that $\Delta I_{\text {trans}}(t)$ remains positive and that $\Delta I_{\text {trans}}(t)$ is zero when either *S*(*t*) or *I*(*t*) is zero; for further discussion, see^30^.

**Table 2:** The mean and standard deviation of the fitted exponents $\gamma$ and $\kappa$, and the correlation between $\gamma$ and $\kappa$, for different network structure.

Params.	$\beta =0.2,\alpha =0.05$	$\beta =0.2,\alpha =0.1$	$\beta =0.3,\alpha =0.05$	$\beta =0.3,\alpha =0.1$
(a) Two-dimensional grid model, with connection to $k=4$ nearest neighbors (i.e., $d=1$ in Eq. (5)).				
$\gamma$	$0.67 \pm 0.04$	$0.70 \pm 0.05$	$0.66 \pm 0.04$	$0.70 \pm 0.04$
$\kappa$	$0.56 \pm 0.43$	$0.06 \pm 0.42$	$0.66 \pm 0.29$	$0.40 \pm 0.24$
$\text {Cor}(\gamma ,\kappa )$	0.73	0.65	0.41	0.61
(b) Two-dimensional grid model, with connection to $k=8$ nearest neighbors (i.e., $d=2$ in Eq. (5)).				
$\gamma$	$0.68 \pm 0.04$	$0.76 \pm 0.05$	$0.66 \pm 0.06$	$0.75 \pm 0.07$
$\kappa$	$0.62 \pm 0.09$	$0.62 \pm 0.08$	$0.58 \pm 0.05$	$0.62 \pm 0.07$
$\text {Cor}(\gamma ,\kappa )$	0.15	0.57	0.64	0.76

Params.	$\beta =0.3,\alpha =0.05$	$\beta =0.3,\alpha =0.1$		
(c) Two-dimensional Gaussian mixture random graph model, with connection to $k=4$ nearest neighbors.				
$\gamma$	$0.62 \pm 0.03$	$0.66 \pm 0.04$		
$\kappa$	$0.43 \pm 0.23$	$0.22 \pm 0.30$		
$\text {Cor}(\gamma ,\kappa )$	0.54	0.60		

The results are obtained from 100 Monte Carlo Simulation of the stochastic model described in “Model: probabilistic SIR on a graph”. The infection parameters $\alpha$ and $\beta$ are assumed to take different values as indicated in the top row of the tables.

Note that $c,\gamma , \kappa$ are random and differ in each realization of the stochastic model. The mean and standard deviation of $\gamma ,\kappa$ over 100 simulations are reported in Tables 2a,b for the cases $d=1$ and $d=2$, respectively. Also, the curve corresponding to the mean values of the parameters is depicted in the second and third panels for comparison with the simulation data points (in orange color). We observe that in all cases, the standard deviation of the fitted exponent $\gamma$ is small, around 0.04. However, the standard deviation over $\kappa$ is large, in some cases around 0.4. This is due to some extent to the fact that the simulations were carried out over short time window, so that the change in *S* is not sufficiently significant to lead to a reliable exponent $\kappa$ (i.e., the fractional change in *S* is relatively small, hence there is an inherent insensitivity to the actual value of the exponent).

As an indicator of the statistical significance of the fractional SIR model, as compared to the traditional model with exponents equal to one, we evaluated the standard model selection *Akaike Information Criterion *(AIC). The AIC score is defined as

7 $$\begin{aligned} \text {AIC} = 2( \# \text {parameters} - \text {max-log-likelihood}), \end{aligned}$$

where the maximum likelihood is obtained by maximizing the probability of observing the data set for the parameters of the model. The assumed fSIR model is

8 $$\begin{aligned} \log (\Delta I_{\text {trans}}(t)) = \log (c) + \gamma \log (I(t)) + \kappa \log (S(t)) + \epsilon (t), \end{aligned}$$

with $\epsilon (t)$ independent Gaussian random noise, for each *t*, having mean zero and unknown variance denoted by $\sigma ^2$. Because of the Gaussian assumption for the noise, the log-likelihood takes the following form

9 $$\begin{aligned} \text {log-likelihood} = -\frac{T}{2}\log (2\pi \sigma ^2) -\sum _{t=1}^T \frac{1}{2\sigma ^2} \left[ \log (\Delta I_{\text {trans}}(t)) - \log (c) - \gamma \log (I(t)) - \kappa \log (S(t))\right] ^2, \end{aligned}$$

where *T* is the number of data-points. In our results, we report the log-likelihood normalized by the number of data points, i.e. $\frac{1}{T} \text {log-likelihood}$.

**Figure 6:**
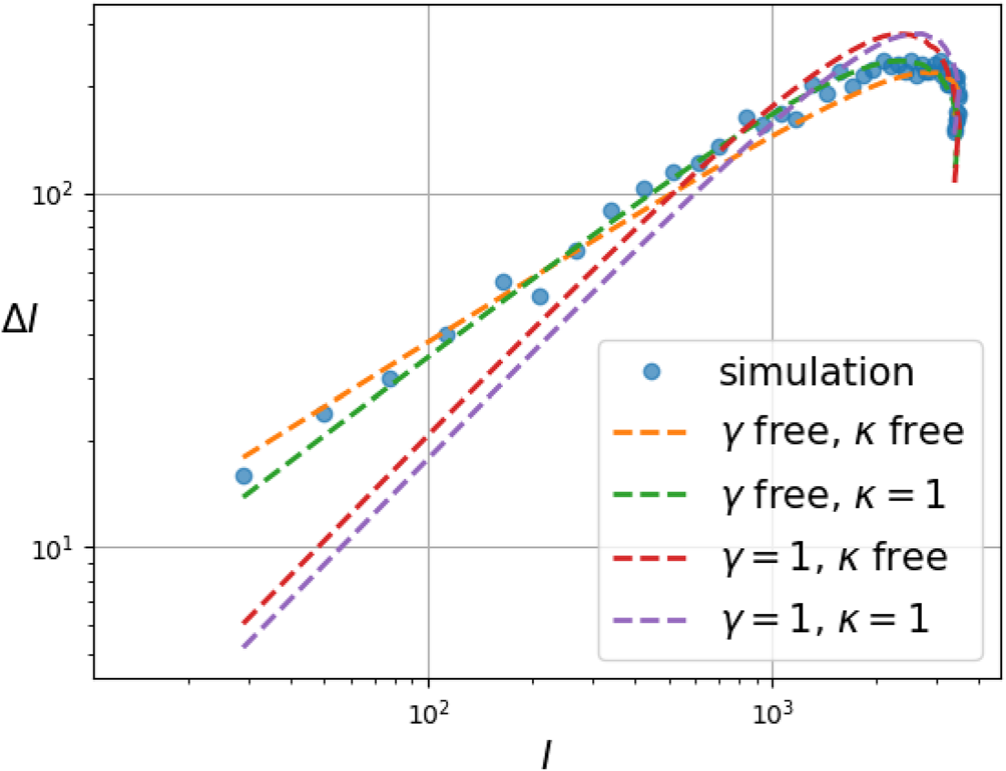
Comparison of four fSIR models with free and partially specified choice of exponents (with the SIR model corresponding to $\gamma =1$ and $\kappa =1$) based on simulation data obtained from two-dimensional grid model with infection parameter $\alpha =0.05$ and $\beta =0.3$. The AIC and maximum likelihood scores for these models are reported in Table 3.

For the purpose of comparison, we consider four different models: (1) our proposed fSIR model where $\gamma$ and $\kappa$ are free, (2) $\gamma$ is free and $\kappa =1$, (3) $\gamma =1$ and $\kappa$ is free, and (4) $\gamma =1$ and $\kappa =1$. The number of unknown parameters for the first model is 4, namely, $c,\gamma ,\kappa ,\sigma$. For the second and the third model, the number of unknown parameters is 3, and for the last one it is 2. We computed the AIC scores and the normalized maximum log-likelihood for all four models averaged over 100 Monte-Carlo simulations for the two-dimensional grid network structure. The results for the setting (Manhattan distance) $d=1$ and $d=2$ are reported in Tables 3a,b, respectively. The reported values suggest a preferance for the fSIR model over models with exponents equal to one; the goodness of the fit more than compensates for the increased “complexity in the model” due to the additional parameters. For illustration purposes, we compare the four models fit to the data in Fig. 6.

**Table 3:** Model selection results for simulated and real data.

Fitting model	$\beta =0.2,\alpha =0.05$		$\beta =0.2,\alpha =0.1$		$\beta =0.3,\alpha =0.05$		$\beta =0.3,\alpha =0.1$	
	AIC	MLL	AIC	MLL	AIC	MLL	AIC	MLL
(a) Two-dimensional grid model, with connection to $k=4$ neighbours (i.e. $d=1$ in (5))								
$$\gamma $$ and $$\kappa $$ free	$$-126.64$$	1.35	$$ -61.38$$	0.69	$$-140.59$$	1.49	$$-104.23$$	1.12
$\gamma$ free $\kappa =1$	$-103.28$	1.09	$-43.37$	0.49	$-75.48$	0.81	$-30.35$	0.36
$\gamma =1$ and $\kappa$ free	7.62	$-0.02$	9.05	$-0.03$	19.26	$-0.13$	21.40	$-0.15$
$\gamma =1$ and $\kappa =1$	35.95	$-0.32$	24.63	$-0.21$	32.19	$-0.15$	23.44	$-0.19$
(b) Two-dimensional grid model, with connection to $k=8$ neighbours (i.e. $d=2$ in (5))								
$$\gamma $$ and $$\kappa $$ free	$$-18.84$$	0.27	$$ -62.87$$	0.71	$$-58.93$$	0.67	$$-82.37$$	0.90
$\gamma$ free $\kappa =1$	51.20	$-0.45$	14.76	$-0.09$	85.41	$-0.79$	63.98	$-0.56$
$\gamma =1$ and $\kappa$ free	54.05	$-0.48$	31.90	$-0.26$	8.03	$-0.02$	21.08	$-0.15$
$\gamma =1$ and $\kappa =1$	60.32	$-0.56$	32.20	$-0.29$	83.58	$-0.80$	64.58	$-0.61$

Fitting model	$\beta =0.3,\alpha =0.05$		$\beta =0.3,\alpha =0.1$					
	AIC	MLL	AIC	MLL				
(c) Two-dimensional Gaussian mixture random graph, with connection to $k=4$ neighbours								
$\gamma$ and $\kappa$ free	$-106.28$	1.14	$-76.82$	0.85				
$\gamma$ free $\kappa =1$	$-1.77$	0.08	$-1.38$	0.31				
$\gamma =1$ and $\kappa$ free	36.68	$-0.31$	36.01	$-0.13$				
$\gamma =1$ and $\kappa =1$	35.80	$-0.32$	21.25	$-0.17$				

Fitting model	Italy		Germany		France		Spain	
	AIC	MLL	AIC	MLL	AIC	MLL	AIC	MLL
(d) Real data from four different countries								
$\gamma$ free	121.33	$-0.81$	156.18	$-1.01$	175.54	$-1.24$	148.34	$-1.09$
$\gamma =1$	216.93	$-1.46$	203.20	$-1.31$	219.55	$-1.55$	204.94	$-1.51$

For the simulated data, the fitting models are based on (8) with parameters $c,\gamma ,\kappa ,\sigma$. The models differ in that the exponents $\gamma$ or $\kappa$ are fixed or optimally selected (free). For the real data, the size of susceptible population *S*(*t*) is assumed to be constant and the exponent $\kappa$ is ignored. The AIC represents the Akaike information criterion defined according to (7) and MLL denotes the normalized maximum log-likelihood computed by maximizing (9) over free parameters. The reported results for the simulated data are averaged over 100 Monte-Carlo simulations. For the simulated data, the infection parameters $\alpha$ and $\beta$ are assumed to take different values as indicated in the top row of the tables.

**Figure 7:**
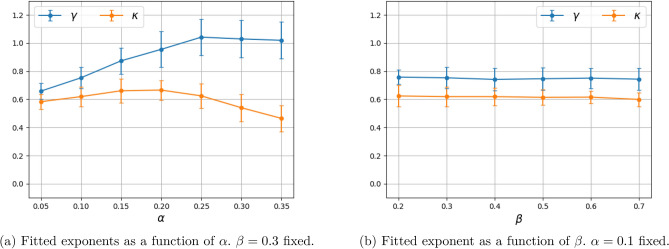
Dependence of the fitted exponents $\gamma$ and $\kappa$ on the model parameters $\alpha$ and $\beta$ for the two-dimensional grid model with $k=4$ nearest neighbors. (Mean and error bars for standard deviation are based on 100 Monte Carlo simulations).

In order to study the effects of model parameters $\alpha$ and $\beta$ on the fitted exponents $\gamma$ and $\kappa$, we carried out additional experiments and reported the results in Fig. 7. Varying $\beta$ does not affect the fitted exponent significantly, however a change in $\alpha$ does. This is consistent with the exponent being impacted by the interface (boundary) between the infected population and susceptible population; a change in $\beta$ affects the speed that the interface propagates. A change in $\alpha$ impacts the interface by changing the rate at which the population inside clusters become susceptible again.

### Two-dimensional random graph

**Figure 8:**
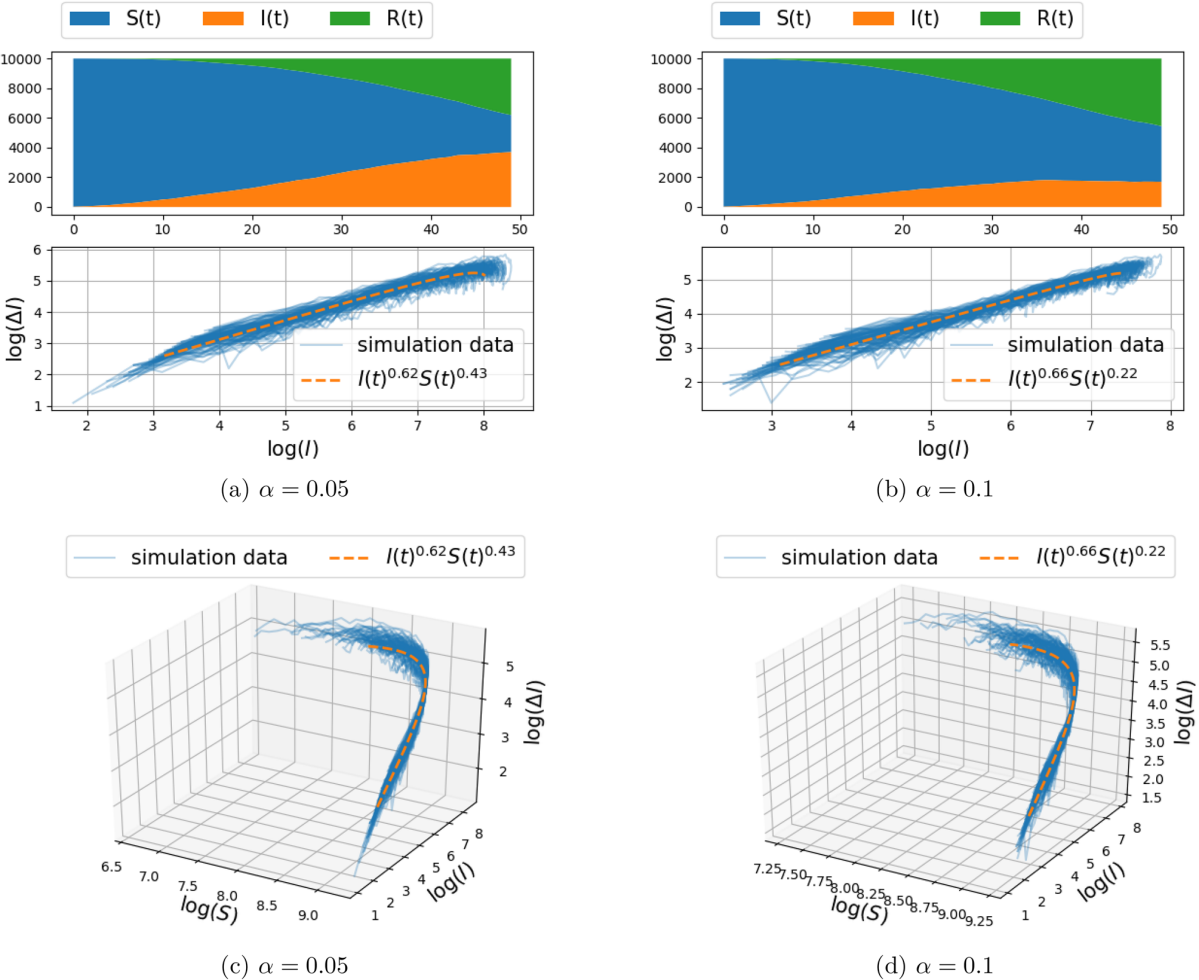
Spread of infection on a mixture of Gaussian random graph model for transmission rate $\beta =0.3$ (the recovery rate $\alpha =0.05$ or $\alpha =0.1$).The top panels depict the number of susceptible, infected, and recovered individuals as a function of time over a range of 50 days, for a single realization of the stochastic model described in “Model: probabilistic SIR on a graph”. The center and bottom panel depict $(\Delta I_{\text {trans}}(t),I(t))$ and $(\Delta I_{\text {trans}}(t),I(t),S(t))$ respectively, for 100 Monte–Carlo simulations (with solid blue lines). The fractional model fit is illustrated with orange dashed curve. The fitting procedure maximizes the log-likelihood (9) over the free parameters. The value of the fitted exponents are shown in the caption.

Simulation result on a two-dimensional random graph model were carried out and documented in Fig. 8. The spatial distribution of $10^4$ node-coordinates ((*x*, *y*)-coordinates, cf. Fig. 1b) has been selected from the mixture of three Gaussians distributions on the node-coordinate plane,

$$\begin{aligned} \frac{1}{3} N\left( \begin{bmatrix} 0 \\ 0 \end{bmatrix}, \begin{bmatrix} 2.0 &{} 0.0\\ 0.0 &{} 0.5 \end{bmatrix} \right) + \frac{1}{3} N\left( \begin{bmatrix} 1 \\ 5 \end{bmatrix}, \begin{bmatrix} 1.44 &{} 0.0\\ 0.0 &{} 0.5 \end{bmatrix} \right) + \frac{1}{3} N\left( \begin{bmatrix} 5 \\ 1 \end{bmatrix}, \begin{bmatrix} 0.64 &{} 0.0\\ 0.0 &{} 1.44 \end{bmatrix} \right) . \end{aligned}$$

Here, *N*(*v*, *R*) denotes a Gaussian distribution with mean *v* and covariance *R*. Initially, for the experiment documented in Fig. 8, the nodes are connected to their 4 nearest neighbors. The infection spread model is once again the one specified in (4). The parameters selected for the results in Fig. 8 are $\beta =0.3$ and $\eta =0.01$, while we compare the effect of $\alpha \in \{0.05,0.1\}$. The numerical result contains 100 independent simulations where each simulation involves independent random construction of the graph according to the Gaussian mixture model, and also random initialization of infected nodes. As before, the first layer of panels depicts the number of susceptible, infected, and recovered individuals as a function of time, for a single realization. The two-dimensional projection of the $(\Delta I(t),I(t),S(t))$ curve on the $(\log (\Delta I(t)), \log (I(t)))$ plane is depicted in the second layer of panels and compared to the distribution of $(\Delta I(t),I(t))$ point set, while the third layer of panels compares the fit of curve to the $(\Delta I(t),I(t),S(t))$ data set in a three-dimensional plot once again in logarithmic scales. The mean and the standard deviation of the fitted exponents are reported in Table 2c. We carry out the model selection procedure, described for the two-dimensional grid, for the random graph and report the results in Table 3c.

**Figure 9:**
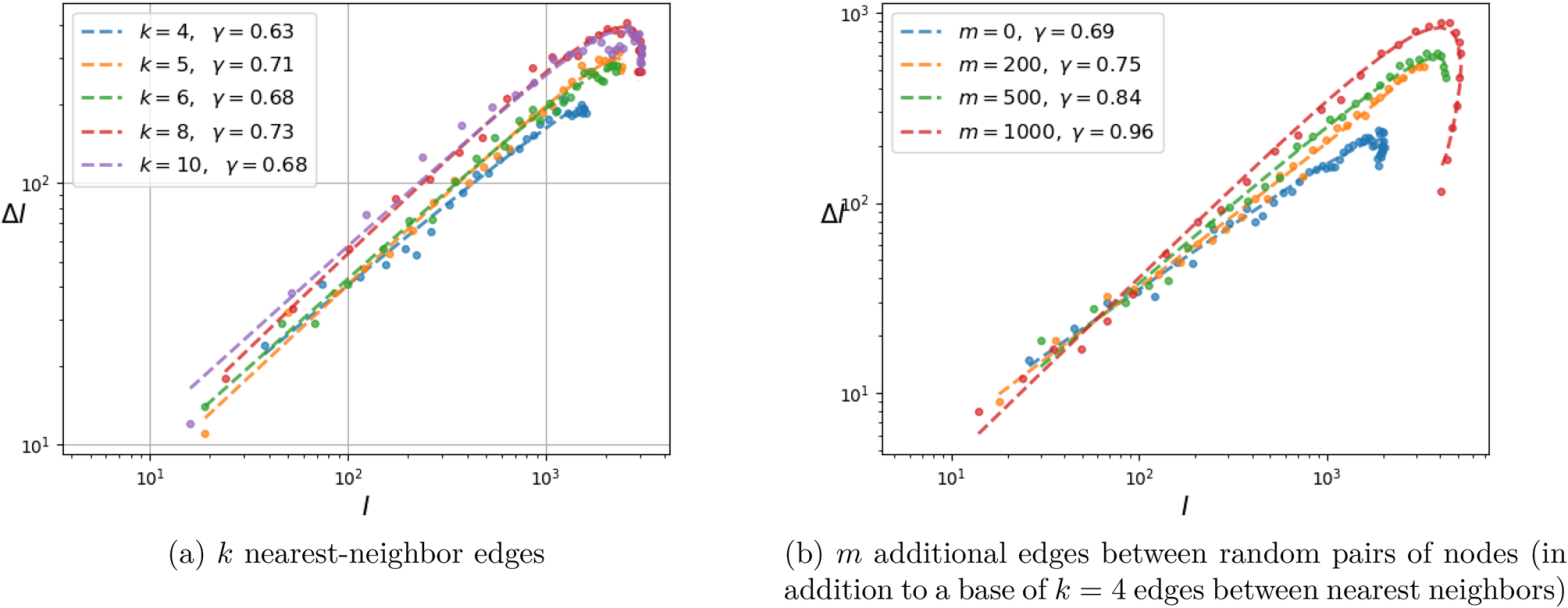
Dependence of the fitted exponent $\gamma$ on graph connectivity for the mixture of Gaussian random graph model. The infection parameters $\alpha =0.1$ and $\beta =0.3$ are fixed. The network parameters, the number of nearest neighbors *k* and the number of random additional edges *m* vary. A single realization is displayed for each choice of network parameters.

**Figure 10:**
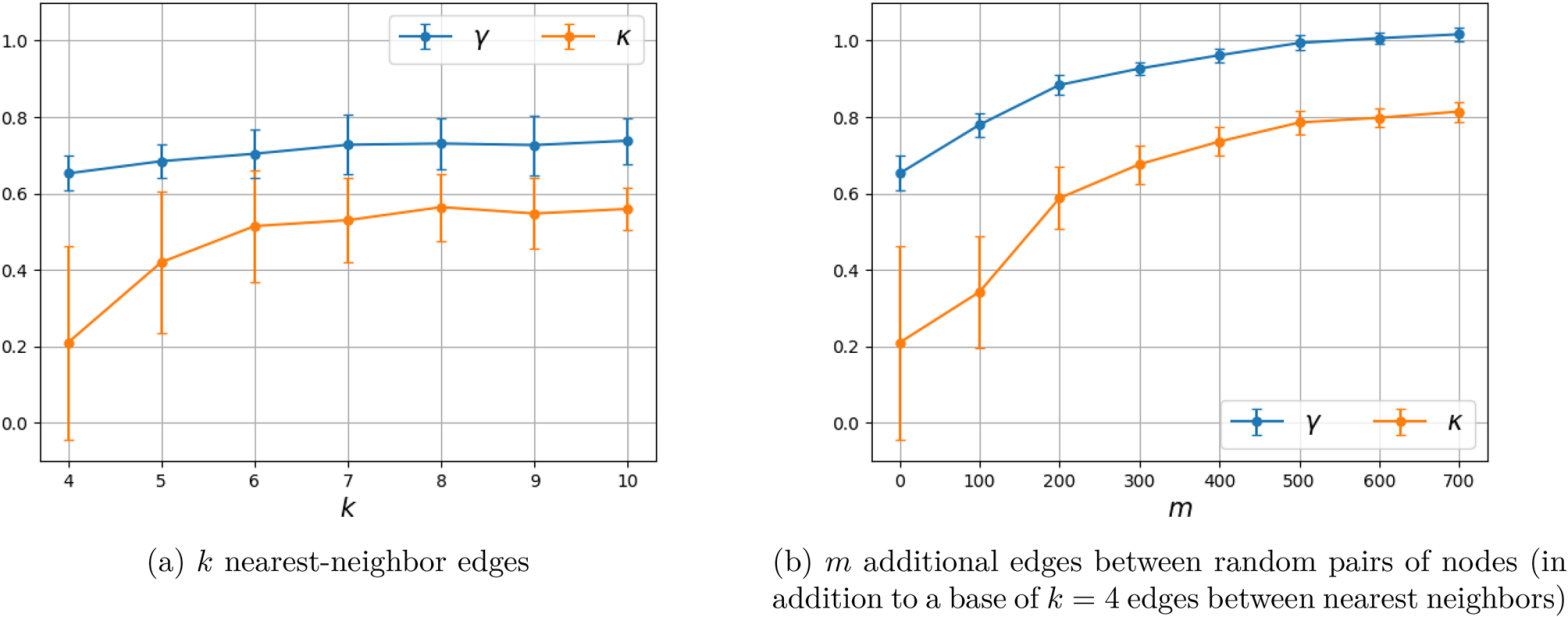
Dependence of the fitted exponents $\gamma$ and $\kappa$ on graph connectivity for the mixture of Gaussian random graph model. The infection parameters $\alpha =0.1$ and $\beta =0.3$ are fixed. The network parameters, the number of nearest neighbors *k* and the number of random additional edges *m* vary. (Mean and error bars for standard deviation are based on 100 Monte Carlo simulations).

Two additional plots that help explain the dependence of $\gamma ,\kappa$ on the geometry of the network are shown in Figs. 9 and 10. Specifically, Fig. 9 shows the result of a single Monte Carlo simulation whereas Fig. 10 displays statistics from 100 simulations. In either figure, the left panel shows the dependence of $\gamma$ (and in addition, $\kappa$ in Fig. 10) as the number *k* of nearest neighbors to each node varies in $\{4,5,6,7,8,9,10\}$. The right panel shows the dependence of the same quantities as additional edges between randomly selected pairs of nodes are introduced. In the figure, $m\in \{0,100,200,300,400,500,600,700\}$ designates the number of additional random edges in this graph of $10^4$ nodes.

It appears, as hypothesized, that the exponent $\gamma$ is affected by the local structure of the graph. If vertices are only connected to nearest neighbors, then $\gamma$ is small. When random connections are introduced (thereby reducing the effective diameter of the graph, i.e., “small world” effect), $\gamma$ increases to 1. Note that only changing the number of connections to a nearest neighbor does not affect the exponent as shown in Fig. 10a.

### COVID-19 data-set

**Figure 11:**
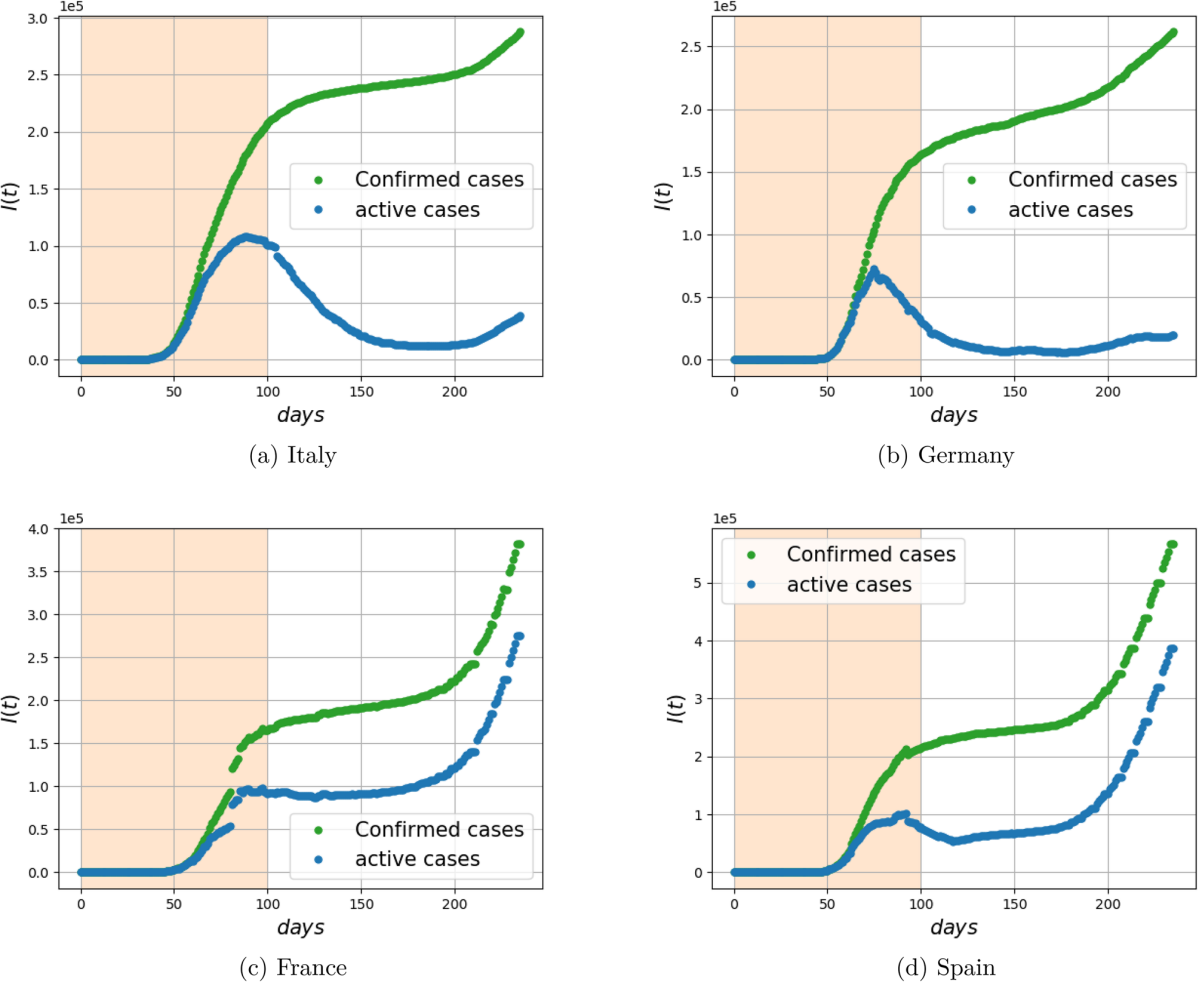
Number of confirmed and active cases in four different countries during the period 01/22/2020 to 09/13/2020. The shaded area is the time period where the fractional model is used to fit the data in Fig. 12. The number of active cases is computed by subtracting the total deaths and recovered from total confirmed cases.

**Figure 12:**
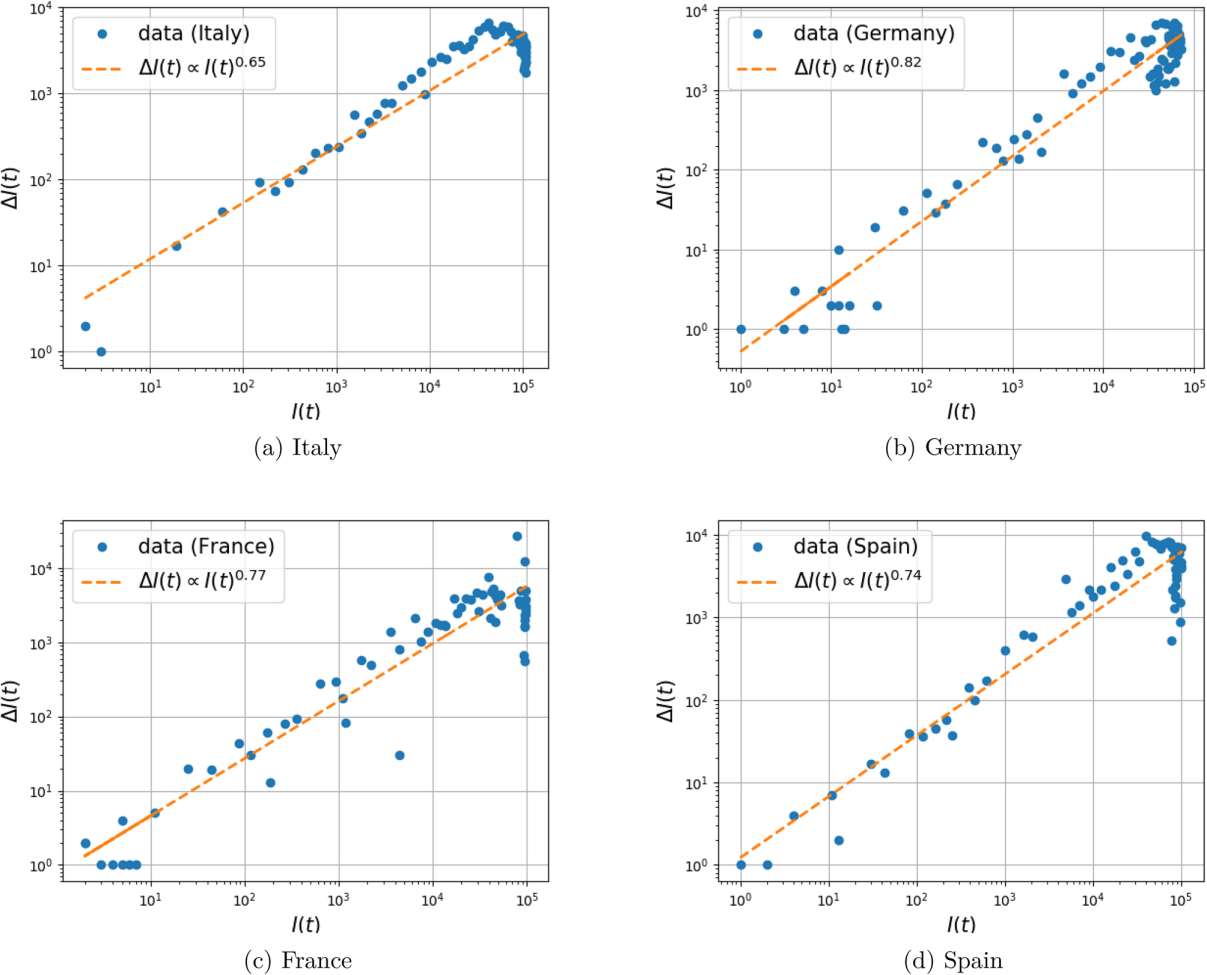
Relationship between infection growth due to transmission $\Delta I_{\text {trans}}(t)$ and the number of infected individuals *I*(*t*) (active cases) with COVID-19 in four different countries. The data belongs to the first 100 days of the infection starting from 01/22/2020.

We utilized the COVID-19 data repository by the center for systems science and engineering (CSSE) at Johns Hopkins University^7,9^. We study the relationship between the number of newly infected $\Delta I_{\text {trans}}(t)$ and the total number of infected individuals *I*(*t*) for the duration January 22 to September 13, in Italy, Germany, France, and Spain. The number of newly infected individuals is available as the number of newly confirmed cases each day. The total number of infected individuals (active cases) is calculated by subtracting the number of deaths and recovered from the cumulative sum of confirmed cases. The time evolution of the number of confirmed cases and active cases, for these four different countries, is depicted in Fig. 11. We fitted the model

10 $$\begin{aligned} \log (\Delta I_{\text {trans}}(t)) = \log (c) + \gamma \log (I(t)) \end{aligned}$$

to the data, for the parameter *c* and exponent $\gamma$, for the first 100 days of the pandemic (starting on January 22). Due to the fact that $S(t)\gg I(t)$ during these initial stages of infection spread, *S*(*t*) is treated as constant. The result for the four different countries is depicted in Fig. 12. The model selection result, in comparison to the model where $\gamma =1$ is fixed, is presented in Table 3d.

## Discussion

The recent onset of the COVID-19 pandemic has underscored the urgency of accurate models for the spread of infectious diseases. These may help guide the allocation of resources, intervention, and mediation strategies, and may help quantify the impact of lifestyle changes on the progression of the epidemic and the threshold for herd immunity^8^. In particular, one immediate practical question in the current COVID-19 pandemic is to decide when an intervention—such as reinstitution of social distancing (after this has been relaxed)—is appropriate. Thus, while efforts to develop accurate models go back almost a century^16^ (see also^4,13^), the subject is especially urgent today.

The main thesis of this work is that models of epidemics, especially during the early phases, incorrectly assume that the contagion depends on the product of infected and susceptible populations. Contagion takes place at the boundary of infected cells and as a result it is the topology of the distribution of infected cells that dictate the spread. An analogous situation takes place in tumor growth, where models suggest (see^3,21,23^) fractional exponent for the contribution of tumor volume, as this more accurately captures the size of the boundary that affects growth. Thus, based on an analogous rationale, we propose a fractional-power alternative, fSIR, to the standard SIR model of disease dynamics. The value of exponents depend on a number of factors including the nature of the boundary between infected cells and the general susceptible population. Specifically, the exponent relates to the level mixing between infected and healthy individuals at interface between the two sub-populations, and may be quantified by the diameter of the graph that represents contacts between individuals.

Our thesis is supported by simulation results as well as by fitting this fSIR model to recent COVID-19 datasets. Specifically, the two-dimensional discrete probabilistic SIR models in Figs. 2 and 3 (with $k=4$ nearest-neighbor connection) and in Figs. 4 and 5 (with $k=8$ nearest-neighbor connection), that simulate disease propagation on a discrete domain of nodes (representing individuals in contact with one another), suggest exponents $\gamma$ in the range between 0.66 and 0.76 for the contribution of *I*(*t*) on the infection rate. Similar results are observed in Fig. 8 for a two-dimensional random distribution of nodes (vertex set) with four nearest-neighbor contacts (edge set). Here, the exponent of the *I*(*t*) contribution to the infection rate lies in a similar range ($\{0.62,0.66\}$ for the conditions displayed). Two additional plots in Fig. 10 highlight the weak dependence of $\gamma$ on the number of short-range contacts (nearest neighbors) and the strong dependence on even a few long-range contacts amongst the general population.

The fit of the COVID-19 data-set^9^ gives exponents for the contribution of *I*(*t*) on the infection rate in the range of 0.6–0.8. In this data-set, the value of *S*(*t*) (that includes the remaining of a rather large total population) varies insignificantly over time, and hence may be treated as constant. A limitation of our experiment is that the value of *I*(*t*) is only an estimate since recording of *all* infected individuals is not guaranteed. Our findings relate to patterns reported in recent studies on COVID-19^11,28^. In particular^28^, reports a power growth law of the infected population with time, which is consistent with our proposal since, in general, fractional exponents lead to power laws (Assuming for simplicity that *S*(*t*) is constant, and the recovery rate $\alpha =0$, then the fSIR equation simplifies to ${\dot{I}} = \beta I(t)^\gamma .$ When $\gamma \in (0,1)$, the solution can be readily expressed as $I(t) = (c_0 + \beta (1-\gamma )t)^{\frac{1}{1-\gamma }}$ for a constant $c_0$. This represents a power law in *t*, unless $\gamma =1$, in which case the solution $I(t) = c_0e^{\beta t}$ is exponential).

An important direction for future research lies in the better understanding of how fractional powers in macroscopic dynamics arise from the probabilistic-network epidemic models. The majority of existing literature on epidemics on networks deals with steady state analysis and is aimed at assessing percolation thresholds and conditions for the appearance of an endemic state (see the review paper^25^). It will be also of great interest to explore connections with a body of literature on reaction diffusion dynamics^6,15,31,32^ for the purpose of gaining insight into macroscopic laws for epidemics.

The authors believe that it is imperative that a deeper and more extensive study is carried out, whereupon the values of $I(t),\Delta I(t), R(t)$ are estimated from more extensive datasets. The effect of mediation efforts, such as social distancing, should be recorded as well and taken into account by differentiating data for the periods before and after such mediation protocols take effect. It is the authors’ hope that questions raised in this work, as to the validity of the basic assumption in SIR models, lead to more reliable and robust ways to estimate the progression of epidemics as well as the progression of the current COVID-19.

## Data Availability

Computer code used in this work is available at https://github.com/AmirTag/fSIR.
